# Effects of pneumococcal conjugate vaccines on reducing the risk of respiratory disease associated with coronavirus infection

**DOI:** 10.1186/s41479-023-00112-w

**Published:** 2023-05-25

**Authors:** Eileen M. Dunne, Marta C. Nunes, Mary P. E. Slack, Christian Theilacker, Bradford D. Gessner

**Affiliations:** 1 Pfizer Vaccines, Collegeville, PA USA; 2 Center of Excellence in Respiratory Pathogens, Hospices Civils de Lyon and Centre International de Recherche en Infectiologie (CIRI) Inserm U1111, CNRS UMR5308, ENS de Lyon, Université Claude Bernard, Lyon 1, Lyon, France; 3 South African Medical Research Council, Vaccines & Infectious Diseases Analytics Research Unit, Faculty of Health Sciences, University of the Witwatersrand, Johannesburg, South Africa; 4 School of Medicine & Dentistry, Griffith University, Gold Coast Campus, Southport, QLD Australia

**Keywords:** Pneumococcal conjugate vaccines, *Streptococcus pneumoniae*, Pneumococcus, Coronaviruses, SARS-CoV-2, COVID-19

## Abstract

Pneumococcal conjugate vaccines (PCVs) provide protection against vaccine-type pneumococcal disease in both children and adults. Growing evidence suggests that PCVs also reduce pneumonia and lower respiratory tract infections (LRTIs) more broadly, including protecting against viral-associated respiratory diseases. In this short narrative review, we highlight clinical studies investigating whether PCVs might have a role in reducing coronavirus disease, both those caused by endemic human coronaviruses (HCoVs) and severe acute respiratory syndrome coronavirus-2 (SARS-CoV-2). These studies include two randomized controlled trials assessing HCoV-associated pneumonia, one each in children and older adults, and two observational studies of PCV13 effectiveness against HCoV-associated LRTI and COVID-19 in adults. We discuss possible mechanisms for PCV protection including preventing viral pneumococcal co-infections and the possibility that pneumococci in the upper respiratory tract might modify the host immune response to SARS-CoV-2. Lastly, we identify knowledge gaps and further questions on the potential role of PCVs during the COVID-19 pandemic.

## Background

Lower respiratory tract infections (LRTIs) remain a major cause of morbidity and mortality worldwide. LTRIs are caused by a wide range of viral, bacterial, and fungal pathogens, some of which also colonize the upper respiratory tract of healthy individuals. *Streptococcus pneumoniae* (pneumococcus) is a leading bacterial cause of community-acquired pneumonia (CAP) in both children and adults [1]. Synergistic interactions between *S. pneumoniae *and respiratory viruses, particularly influenza virus, are well-documented [2]. Pneumococcal conjugate vaccines (PCVs) protect against disease caused by vaccine-type pneumococci and also reduce pneumococcal colonization of the upper respiratory tract. In both children and adults, evidence from randomized clinical trials (RCTs) and epidemiological studies suggests that PCVs might reduce viral lower respiratory tract disease in addition to pneumococcal disease [3, 4].

Coronaviruses (CoVs) are enveloped RNA viruses. Endemic human coronaviruses (HCoVs) 229E, NL63, OC43, and HKU1 typically cause mild, seasonal upper respiratory tract illness, but can cause LRTIs, especially in older adults and people with co-morbidities [5]. Three epidemic CoVs have emerged since the early 2000s, most recently severe acute respiratory syndrome coronavirus-2 (SARS-CoV-2), the cause of the coronavirus disease 2019 (COVID-19) pandemic. Early during the COVID-19 pandemic, researchers explored whether existing vaccines could provide protection against coronaviruses including SARS-CoV-2. Now that safe and effective COVID-19 vaccines are available, this question remains interesting from scientific and public health perspectives. It is not known whether combining COVID-19 vaccines with other vaccines could lead to better protection, for example longer protection or cross-protection against SARS-CoV-2 variants. In this minireview, we provide a concise overview of evidence on PCVs and coronavirus infections from randomized clinical trials and effectiveness studies, discuss potential mechanisms, and highlight evidence gaps.

## Main text

As part of a comprehensive systematic literature review on PCVs and viral respiratory infections, registered in the PROSPERO database (registration number: CRD42022339625), we identified four clinical studies investigating the efficacy or effectiveness of PCV against HCoVs or COVID-19 (Table 1). No other relevant studies were identified.

**Table 1:** Summary of randomized controlled trials and observational studies investigating pneumococcal conjugate vaccine efficacy or effectiveness against coronaviruses

**Reference**	**Vaccine**	**Study population**	**Outcome**	**Vaccine efficacy or effectiveness** **% (95% CI),** ***P*** **value**^a^
Randomized controlled trials				
Nunes 2021 [6]	PCV9	HIV-uninfected children aged < 2y in South Africa	HCoV-associated pneumonia (any)	64.0 (22.9–83.2), *P* = 0.006
			HCoV-associated pneumonia (single viral infection)	50.0 (-66.0–84.9), *P* = 0.25
			HCoV-OC43-associated pneumonia	90.9 (29.5–98.8), *P* = 0.004
			HCoV-HKU1-associated pneumonia	87.5 (0.1–98.4), *P* = 0.020
			HCoV-NL63-associated pneumonia	-33.3 (-248.1–53.7), *P* = 0.59
Huijts 2018 [7]	PCV13	Adults ≥ 65y in The Netherlands	HCoV-associated confirmed CAP	23.6 (99.3%CI -54.3–62.2), *P* = 0.302
			HCoV-associated suspected pneumonia	30.6 (99.3%CI -14.0–57.8), *P* = 0.047
Observational studies				
Lewnard 2022 [4]	PCV13	Adults ≥ 18y in the US	*Any virus detected*^*c*^	
			HCoV-associated LRTI or pneumonia^d^	27.4 (4.5–44.7)
			HCoV-associated LRTI (non-pneumonia)	51.4 (6.0–74.9)
			HCoV-associated pneumonia	23.5 (-4.1–43.8)
			Hospitalized HCoV-associated LRTI or pneumonia	19.9 (-13.1–43.2)
			Non-hospitalized HCoV-associated LRTI or pneumonia	38.7 (2.6–61.4)
			*Single virus detected*	
			HCoV-associated LRTI or pneumonia	31.5 (5.9–50.1)
			HCoV-associated LRTI (non-pneumonia)	55.3 (-12.9–82.3)
			HCoV-associated pneumonia	32.7 (4.8–52.5)
			Hospitalized HCoV-associated LRTI or pneumonia	32.5 (-1.2–55.0)
			Non-hospitalized HCoV-associated LRTI or pneumonia	40.8 (-2.7–65.9)
Lewnard 2021 [8]	PCV13	Adults ≥ 65y in the US	COVID-19 diagnosis	35 (28–72)^b^
			COVID-19 hospitalization	32 (17–43)^b^
			Fatal COVID-19 hospitalization	32 (5–51)^b^

*PCV* Pneumococcal conjugate vaccine, *HIV* Human immunodeficiency virus, *HCoV* Endemic human coronavirus, *CAP* Community-acquired pneumonia, *LRTI* Lower respiratory tract infection, *COVID-19* Coronavirus disease 2019

^a^P values were only reported for randomized controlled trials

^b^Vaccine effectiveness calculated as 1 – adjusted hazard ratio × 100%

^c^The following viruses were tested for: influenza A and B viruses, respiratory syncytial virus, HCoVs (including the 229E, HKU1, NL63, and OC43 subtypes), parainfluenza viruses (types 1–4), adenoviruses, human metapneumovirus, and enteroviruses (including rhinoviruses)

^d^LRTI and pneumonia cases were defined according to codes from the International Classification of Diseases, Tenth Revision, Clinical Modification

### Clinical trials assessing PCV efficacy against HCoVs

An RCT in South African infants demonstrated that an experimental PCV9 prevented 31% (95% confidence intervals[CI] 15 to 33%) of viral-associated pneumonia among hospitalized children [3]. A post-hoc analysis of this trial reported that PCV9 reduced pneumonia associated with HCoV in children < 2 years old (vaccine efficacy [VE], 33.9%; 95%CI 2.0 to 55.4%), especially in HIV-uninfected (VE, 64.0%; 95%CI 22.9 to 83.2) (Table 1), but not in HIV-infected children (VE, 13.6%; 95%CI -37.8 to 45.8) [6]. Protection against individual HCoVs in HIV-uninfected children varied, with high efficacy against HCoV-OC43 and HCoV-HKU1, but no protection for CoV-NL63 (Table 1). An exploratory analysis of an RCT in older Dutch adults using conservative 99.3% CIs reported that PCV13 did not significantly reduce HCoV-associated CAP (Table 1) [7]. For HCoV-associated suspected pneumonia, the *P* value was < 0.05, however 95%CIs were wide, spanning zero.

### Observational studies evaluating PCV13 effectiveness against HCoVs and COVID-19

In a case–control study, Lewnard et al. evaluated PCV13 effectiveness against virus-associated LRTIs among adults in California, United States during the pre-COVID-19 pandemic period [4]. Matched, adjusted effectiveness against HCoV-associated LRTI was evaluated for several outcomes (Table 1). Significant protection was observed against LRTI or pneumonia when HCoV was detected. For outcome subcategories, all effectiveness point estimates were consistent with protection, but some 95% CIs overlapped zero.

Lewnard and colleagues also investigated PCV13 effectiveness against COVID-19 outcomes in a cohort of older adults in California during 2020 [8]. To reduce potential confounding, analyses included adjustments for multiple covariates and accounted for zoster vaccination as a negative-control exposure. PCV13 displayed significant effectiveness against COVID-19 diagnosis, hospitalization, and mortality (Table 1). In contrast, the pneumococcal polysaccharide vaccine PPV23 did not display protection against COVID-19, suggesting that reduced risk of COVID-19 in PCV13-vaccinated adults might be related to pneumococcal carriage, since, unlike PCV13, PPV23 does not affect pneumococcal carriage [8]. PCV13 effectiveness was abrogated by recent prior antibiotic usage, supporting a role of pneumococcus in the causal chain [8].

### Potential explanations for clinical study findings

In both children and adults, the limited available evidence raises the possibility that PCVs reduce the risk of HCoV-associated respiratory disease. These results indicate that pneumococci might play a role in the development or severity of HCoV infections. Some of the observed efficacy might be due to prevention of pneumococcal-HCoV co-infection, particularly for the PCV9 trial. Since there are no suitable diagnostics for identifying non-bacteremic pneumococcal pneumonia in children, some of the HCoV-associated pneumonia cases may have been pneumococcal-HCoV co-infections for which only HCoV was detected, and these would be more likely to occur in the children who did not receive PCV. Pneumococcal pneumonia is also underdectected in adults due to limited sensitivity of blood culture and urinary antigen tests, so some of the reductions in coronavirus-associated disease in adults might be due to fewer pneumococcal co-infections [9]. Additionally, pneumococcal-viral interactions induce immune responses in the nasopharynx that influence the pathogenesis of respiratory infections [10, 11]. Studies in children and adults have found that pneumococcal densities in the nasopharynx are higher when a respiratory virus or influenza-like illness is present [12, 13]. Higher pneumococcal density in asymptomatic children was associated with increased risk of subsequent acute respiratory illness [13].

In adults, observational studies have reported reduced risk of COVID-19 after vaccination with other vaccines including influenza vaccines and recombinant zoster vaccine [14, 15]. Proposed mechanisms include nonspecific immune activation or confounding due to the ‘healthy vaccinee’ effect, whereby people who get vaccinated tend to be healthier than those who do not, and adjusted analyses fail to fully overcome underlying differences between vaccinated and unvaccinated participants. In a placebo-controlled RCT, bacille Calmette-Guérin (BCG) vaccination failed to protect older adults against SARS-CoV-2 respiratory infection, making the hypothesis of ‘trained immunity’ as the basis of heterologous vaccine protection less likely [16]. The elimination of benefit with prior antibiotics in the California PCV13 observational study makes this hypothesis less likely for PCV13 [8].

For PCV13, another plausible mechanism exists: the observed reduction in COVID-19 risk might be due to pneumococcal carriage modifying host immune responses [8]. In a study conducted among healthcare workers and patients, pneumococcal colonization was associated with diminished antiviral immune responses to SARS-CoV-2 in both cohorts [17]. SARS-CoV-2-specific salivary IgA in healthcare workers and SARS-CoV-2-specific memory B-cells in patients were lower in pneumococcal-colonized subjects compared to those without pneumococcal colonization [17]. These results build upon human challenge models exploring the relationships between pneumococcal colonization, nasopharyngeal microbiota, and responses to live attenuated influenza vaccine [11]. Typically, viruses are thought to precipitate secondary pneumococcal infections, however surveillance data from the UK raises the possibility that pneumococcal infection might increase risk or severity of subsequent COVID-19 [18, 19]. A study in US adults demonstrated higher adjusted odds of SARS-CoV-2 infection (2.73, 95%CI 1.58 to 4.69) among pneumococcal carriers and positive associations between SARS-CoV-2 and indicators of pneumococcal density, further suggesting synergistic interactions [20]. Together, these data raise the hypothesis that PCVs might reduce secondary infections caused by coronaviruses.

### Limitations, remaining scientific questions, and data gaps

The clinical studies included in our summary have their own inherent limitations and biases. The two RCTs were not designed to evaluate coronavirus outcomes, and post-hoc or exploratory analyses should be interpreted with caution. The detection of HCoVs in children with pneumonia in the PCV9 trial is insufficient to demonstrate causality, however the reductions observed in the vaccine arm, plus consistency with the finding that efficacy against vaccine-type invasive pneumococcal disease was greater in HIV-uninfected children, suggest that PCV9 protects against HCoV-pneumococcal co-infection [21]. All observational studies are subject to bias, and although the two observational studies attempted to reduce bias through adjusted analyses and either matching [4] or correction using a negative control exposure [8], some residual bias, particularly due to the ‘healthy vaccinee’ effect, may remain [22].

The potential impact of PCVs on disease due to coronaviruses, including SARS-CoV-2, remains unknown. PCV9 efficacy against HCoV-associated pneumonia seen in South African children may not apply to SARS-CoV-2, as protection against individual HCoVs varied, and HCoVs primarily affect the upper respiratory tract whilst SARS-CoV-2 tropism is broader [6]. High-quality observational studies assessing PCV effectiveness against COVID-19 in children might be difficult to conduct in countries with high PCV uptake through pediatric national immunization programs. Nonetheless, future pediatric studies could explore the relationship between SARS-CoV-2, pneumococcal colonization and density, and respiratory infections.

In adults, evidence of PCV13 effectiveness against COVID-19 stems from a single cohort study conducted prior to COVID-19 vaccine availability [8]. Current data leave many questions unanswered in an era with effective SARS-CoV-2 vaccines. For example, it is unknown whether PCVs could supplement COVID-19 vaccines by reducing vaccine failures occurring because of waning host responses or SARS-CoV-2 variants. Observational studies investigating whether COVID-19 vaccines can prevent pneumococcal disease outcomes could further explore potential relationships between these two pathogens. We do not know of data to indicate whether vaccines against SARS-CoV-2 might also reduce pneumococcal outcomes; however, such an effect is plausible since it is well-known that viruses such as influenza can cause secondary pneumococcal pneumonias. Additionally, no data exist on whether interactions with coronaviruses vary by pneumococcal serotype. There is evidence from studies of other respiratory viruses (respiratory syncytial virus in children and influenza in adults) that viral interactions with pneumococci can differ by serotype [23, 24]. If PCVs have a specific effect on coronavirus disease, we hypothesize that this would be mediated through the reduction of vaccine-type pneumococci, either by reducing co-infection between vaccine-type pneumococci and coronaviruses, or by reducing carriage of vaccine serotypes that may play a role in the causal chain of coronavirus disease. A current gap in understanding is how PCVs might potentiate these effects in settings like the United States where the identification of vaccine-type pneumococci has been relatively low (either because of low carriage prevalence, low density carriage, or inadequate testing methodology), in contrast to the South African PCV9 trial conducted prior to routine use of PCV. Recent evidence indicates that pneumococcal carriage in adults is underestimated, and use of molecular methods, multiple sample types, and longitudinal sampling substantially increases detection of pneumococci, including PCV13 serotypes [25, 26]. Lastly, while pneumococcal carriage leads to changes in host antiviral immune responses, these changes have not been linked to clinical outcomes.

Because PCVs in adults are not widely used, the opportunity exists for high-quality observational studies that incorporate design elements to mitigate risk of bias to address some remaining questions [21]. In vitro and in vivo models could be used to explore the role of serotype in pneumococcal-coronavirus interactions. Extending human challenge models offers another efficient pathway for further research.

## Conclusions

Although available data are limited, there is emerging evidence that PCVs might reduce the risk of respiratory disease caused by coronaviruses including SARS-CoV-2. Future studies can further explore the relationship between pneumococci, coronaviruses, and host immune responses, and the mechanisms by which PCVs might alter these interactions. Understanding the broader impact of PCVs can help optimize vaccination strategies to prevent respiratory infections.

## Data Availability

Not applicable.
